# Supplementary material for: Feasibility trial of Darwin OncoTreat and OncoTarget precision medicine testing to improve outcomes for patients with limited metastatic disease that failed first-line systemic therapy
Source: PLoS One. 2025 Jun 11;20(6):e0325769. doi: 10.1371/journal.pone.0325769 (PMC12157055; doi:10.1371/journal.pone.0325769)
Supplement: Attachment — Submitted filename: Response to Reviewers.docx [file pone.0325769.s003.docx]

PLOS ONE PONE-D-24-45282: Feasibility Trial of Darwin OncoTreat and OncoTarget Precision Medicine Testing to Improve Outcomes for Patients with Limited Metastatic Disease that Failed First-Line Systemic Therapy

**Thank you so much for your thoughtful review of our manuscript. Below is a point-by-point response to all comments we have received.**

**We have additionally added another author to the manuscript. A “Request for Authorship Changes” form and written consent from all coauthors has been uploaded to our revision submission.**

1. Methodology: Include additional statistical considerations for sample size calculation, the study population, and outcome estimates. Please refer to the FDA Draft Guidance for Industry on Master Protocols in Oncology Drugs and Biologics to ensure alignment with recommended practices.

**Response: Additional statistical considerations have been added, including rationale for the sample size and the consideration of subgroup analyses (number of active tumors, type of primary tumor) to evaluate feasibility outcomes. We will provide hazard ratios with 95% confidence intervals and a log-rank test to compare progression-free survival and overall survival between groups.**

2. Protocol Requirements: Review and report all necessary elements from the SPIRIT checklist. Please also ensure the manuscript adheres to the PLOS ONE publication criteria for protocols, as outlined in the Submission Guidelines. Also, please provide background information on the comparative efficacy of OncoTreat and OncoTarget platforms in patients with cancer.

**Response: The comparative efficacy of OncoTarget and OncoTreat is limited, but the findings from a small trial conducted at Columbia University have been included in the background section. This limited literature underscores the value of our trial for the application of OncoTarget and OncoTreat to clinical settings.**

Journal Requirements:

When submitting your revision, we need you to address these additional requirements.

1. Please ensure that your manuscript meets PLOS ONE's style requirements, including those for file naming. The PLOS ONE style templates can be found at
<https://journals.plos.org/plosone/s/file?id=wjVg/PLOSOne_formatting_sample_main_body.pdf> and
<https://journals.plos.org/plosone/s/file?id=ba62/PLOSOne_formatting_sample_title_authors_affiliations.pdf>

**Response: Style requirements have been addressed based on PLOS One’s criteria.**

2. Please ensure you have included the registration number for the clinical trial referenced in the manuscript.

**Response: The clinical trial number is referenced in Methods section under the subtitle “Study design and oversight.” The URL for the trial has been added to the References.**

3. Your ethics statement should only appear in the Methods section of your manuscript. If your ethics statement is written in any section besides the Methods, please move it to the Methods section and delete it from any other section. Please ensure that your ethics statement is included in your manuscript, as the ethics statement entered into the online submission form will not be published alongside your manuscript.

**Response: The ethics statement is now in the Methods section under “Study design and oversight” (last paragraph). The SPIRIT checklist has been updated accordingly.**

4. Your abstract cannot contain citations. Please only include citations in the body text of the manuscript, and ensure that they remain in ascending numerical order on first mention.

**Response: Citations are not present within the abstract and follow sequential order within the manuscript text.**

5. Please include captions for your Supporting Information files at the end of your manuscript, and update any in-text citations to match accordingly. Please see our Supporting Information guidelines for more information: <http://journals.plos.org/plosone/s/supporting-information>.

**Response: Fig 1, Fig 2, and Fig 3 captions are provided within the body, where figures are indicated to go. No further supporting information files are included.**

6. Please review your reference list to ensure that it is complete and correct. If you have cited papers that have been retracted, please include the rationale for doing so in the manuscript text, or remove these references and replace them with relevant current references. Any changes to the reference list should be mentioned in the rebuttal letter that accompanies your revised manuscript. If you need to cite a retracted article, indicate the article’s retracted status in the References list and also include a citation and full reference for the retraction notice.

**Response: To the best of our knowledge the reference list is complete and correct.**

Reviewers' comments:

Reviewer's Responses to Questions

**Comments to the Author**

1. Does the manuscript provide a valid rationale for the proposed study, with clearly identified and justified research questions?
The research question outlined is expected to address a valid academic problem or topic and contribute to the base of knowledge in the field.

Reviewer #1: Yes

Reviewer #2: Yes

**Response: Thank you for your review.**

2. Is the protocol technically sound and planned in a manner that will lead to a meaningful outcome and allow testing the stated hypotheses?

The manuscript should describe the methods in sufficient detail to prevent undisclosed flexibility in the experimental procedure or analysis pipeline, including sufficient outcome-neutral conditions (e.g. necessary controls, absence of floor or ceiling effects) to test the proposed hypotheses and a statistical power analysis where applicable. As there may be aspects of the methodology and analysis which can only be refined once the work is undertaken, authors should outline potential assumptions and explicitly describe what aspects of the proposed analyses, if any, are exploratory.

Reviewer #1: Partly

Reviewer #2: Yes

**Response: Thank you for your review. We believe we have addressed these concerns through the later comments and suggestions.**

3. Is the methodology feasible and described in sufficient detail to allow the work to be replicable?
Descriptions of methods and materials in the protocol should be reported in sufficient detail for another researcher to reproduce all experiments and analyses. The protocol should describe the appropriate controls, sample size calculations, and replication needed to ensure that the data are robust and reproducible.

Reviewer #1: Yes

Reviewer #2: Yes

**Response: Thank you for your review.**

4. Have the authors described where all data underlying the findings will be made available when the study is complete?
The [PLOS Data policy](https://nam10.safelinks.protection.outlook.com/?url=https%3A%2F%2Ftrack.editorialmanager.com%2FCL0%2Fhttps%3A%252F%252Fjournals.plos.org%252Fplosone%252Fs%252Fmaterials-and-software-sharing%2F1%2F010f019448616fc6-a74c3a37-cb83-48c5-9206-5048705a8b35-000000%2F_Gjq1l2jIQVqbv5Y-3jld2tGk9qKCtFMj76ndAqlQzc%3D192&data=05%7C02%7Crradigan%40nyit.edu%7Cba9d4c71472a48f9d3dc08dd30415645%7C1a561373a3a14beda7c281410491a214%7C0%7C0%7C638719780009243443%7CUnknown%7CTWFpbGZsb3d8eyJFbXB0eU1hcGkiOnRydWUsIlYiOiIwLjAuMDAwMCIsIlAiOiJXaW4zMiIsIkFOIjoiTWFpbCIsIldUIjoyfQ%3D%3D%7C0%7C%7C%7C&sdata=QWfWLBn1iHG0H9lbFjPz65ZEbSlqMOWJgYRUAnnBD0E%3D&reserved=0) requires authors to make all data underlying the findings described in their manuscript fully available without restriction, with rare exception, at the time of publication. The data should be provided as part of the manuscript or its supporting information, or deposited to a public repository. For example, in addition to summary statistics, the data points behind means, medians and variance measures should be available. If there are restrictions on publicly sharing data—e.g. participant privacy or use of data from a third party—those must be specified.

Reviewer #1: No

Reviewer #2: Yes

**Response: Thank you for your review. Data will be available through Clinical Trials registration.**

5. Is the manuscript presented in an intelligible fashion and written in standard English?
PLOS ONE does not copyedit accepted manuscripts, so the language in submitted articles must be clear, correct, and unambiguous. Any typographical or grammatical errors should be corrected at revision, so please note any specific errors here.

Reviewer #1: Yes

Reviewer #2: Yes

**Response: Thank you for your review.**

6. Review Comments to the Author

Please use the space provided to explain your answers to the questions above and, if applicable, provide comments about issues authors must address before this protocol can be accepted for publication. You may also include additional comments for the author, including concerns about research or publication ethics.
You may also provide optional suggestions and comments to authors that they might find helpful in planning their study.

(Please upload your review as an attachment if it exceeds 20,000 characters)

Reviewer #1: This manuscript sets out the protocol for evaluating the feasibility of a novel genomic RNA test to aid precision medicine decisions in treating metastatic cancer. The authors describe the rationale well and designed a trial to assess the possibility of integrating the precision medicine test into the standard care pathway. I appreciate that this is an early stage trial and including patients with metastatic cancer is challenging. Please see my suggestions for improving the manuscript. I hope you find this helpful.

1. Please can you explain why there is no ongoing oversight for this trial. The SPIRIT checklist is NA for sponsor and committees for example. If these are not required for this trial design, please include the rationale for this on the SPIRIT checklist, or if proxies for this oversight are available (i.e IRB or PI responsibility).

**Response: Good Samaritan University Hospital IRB is overseeing this trial. Additionally, the trial has been PRS-approved and is currently available to view on ClinicalTrials.gov (NCT06842030). This has been mentioned in the SPIRIT checklist.**

2. Can you give some more justification or rationale for the sample size target of 5-20 patients. Is this in relation to how many patients this institution cares for, for example? Can you reasonably expect meaningful statistical results from 5 patients?

**Response: Due to the nature of our patient population and our feasibility trial design, 20 patients were set to determine feasibility outcomes of the Darwin OncoTarget and OncoTreat test. It is typical for the first phase of a Simon two-stage design to enroll less than 30 patients (**<https://pmc.ncbi.nlm.nih.gov/articles/PMC8688307/>). **An interim analysis of 5 is to ensure continued benefice to patients. Our numbers are also based on a prior feasibility study of only 7 patients and we hope to expand this to gather further information about practicality within a community hospital setting.**

3. Can you include in the supplementary materials the baseline patient and clinical characteristics and endpoint measures, for example, in a simple proforma – presumably you are collecting this information somehow? And when these assessments are made, if at different times during the trial.

**Response: Baseline clinical characteristics will be collected prior to biopsy and precision medicine testing; the specific data elements are detailed in Study Design. Post-test endpoint measures include feasibility of completing the test, changing treatment regimen, procuring prescribed drugs through insurance, and measuring progression-free survival. These endpoints along with toxicities are explained in the Study Design and OncoTarget and OncoTreat Treatment Recommendations.**

a. Also, in Fig1, I am not clear what Q is in Q3 months and Q6 months.

**Response: Thank you for pointing this out. Abbreviated Q for “every”. Updated Fig 1 to avoid abbreviation and make state “every 3 months” and “every 6 months”.**

4. Can you make the following methods clearer…
a. (line 161 & Fig1) What happens to those patients with samples <50% tumor?

**Response: Thank you for your comment. This is one of our feasibility outcomes: 1) ability to have the OncoTarget and OncoTreat test performed based on tumor type and pathology with sufficient cellularity/DNA/RNA; as testing will not be able to be performed on those whose tumor tissue biopsies contain less than 50% of tumor. We had added the latter statement to clarify.**

b. (lines 159-165) can you make it clearer who is doing the biopsy and that Columbia University Medical Center is doing the Darwin testing. I think this is clearer in Fig 3, than it is in the text.

**Response: Thank you for pointing this out. We have added a clarifying statement.**

c. Are you going to report how many patients were ineligible for patient selection overall and why (e.g. no consent, NEAT score)? This would also be useful to know for patient acceptability – e.g. out of how many patients >18 with up to 10 lesions and classify as oligometastases, were included in the trial.

**Response: If there are patients who initially consent to the trial but do not ultimately receive Darwin testing results, the reason for attrition will be reported (eg, voluntary withdrawal, insufficient tumor, death, etc.). Additionally, identifying any unknown barriers to testing is one of our feasibility endpoints. The overall potential pool of patients is those with metastatic cancer referred to radiation oncology, but most of these patients don’t qualify because they have >10 lesions, a NEAT score that’s too low, or they haven’t failed first-line systemic therapy. We’re not including these ineligible patients because they haven’t signed a consent form and therefore are not formally part of the study.**

5. Would the long term goal be for an RCT to evaluate the efficacy of precision medicine using the Darwin tests for metastatic cancer survival? If so, would it be useful to include the acceptability of randomisation to precision medicine, in theory, for both patients and clinicians?

**Response: Thank you for your comment. The long-term goal, with success of this feasibility trial is an RCT. We have added a reflection on this to our Discussion section.**

Reviewer #2: Very Minor Thought
This appears to be a well-designed feasibility trial with the feasibility objectives to be correctly summarized without formal statistical analysis. As the authors will no doubt concede, statistical significance is unlikely when the suggested comparisons of progression free survival and overall survival are made. However, 95%CI intervals for the HRs estimated would be informative.

**Response: That you so much for this added value. We have added 95% CI for HR to our statistical analysis section.**

7. PLOS authors have the option to publish the peer review history of their article ([what does this mean?](https://nam10.safelinks.protection.outlook.com/?url=https%3A%2F%2Ftrack.editorialmanager.com%2FCL0%2Fhttps%3A%252F%252Fjournals.plos.org%252Fplosone%252Fs%252Feditorial-and-peer-review-process%2523loc-peer-review-history%2F1%2F010f019448616fc6-a74c3a37-cb83-48c5-9206-5048705a8b35-000000%2FNubuCtwpMSFWv1jD2c4T6N4Mov-5P7Z6LRorP9_vAK4%3D192&data=05%7C02%7Crradigan%40nyit.edu%7Cba9d4c71472a48f9d3dc08dd30415645%7C1a561373a3a14beda7c281410491a214%7C0%7C0%7C638719780009262070%7CUnknown%7CTWFpbGZsb3d8eyJFbXB0eU1hcGkiOnRydWUsIlYiOiIwLjAuMDAwMCIsIlAiOiJXaW4zMiIsIkFOIjoiTWFpbCIsIldUIjoyfQ%3D%3D%7C0%7C%7C%7C&sdata=0XQdOWW165UBKvm%2FmaZwlvqsp7wJ%2Fd8eOypA24G0i24%3D&reserved=0)). If published, this will include your full peer review and any attached files.
If you choose “no”, your identity will remain anonymous but your review may still be made public.
Do you want your identity to be public for this peer review? For information about this choice, including consent withdrawal, please see our [Privacy Policy](https://nam10.safelinks.protection.outlook.com/?url=https%3A%2F%2Ftrack.editorialmanager.com%2FCL0%2Fhttps%3A%252F%252Fwww.plos.org%252Fprivacy-policy%2F1%2F010f019448616fc6-a74c3a37-cb83-48c5-9206-5048705a8b35-000000%2FLmtC6E_uplLg92YZqEvpzKPwNhsABA9IsFZIpjZ4lao%3D192&data=05%7C02%7Crradigan%40nyit.edu%7Cba9d4c71472a48f9d3dc08dd30415645%7C1a561373a3a14beda7c281410491a214%7C0%7C0%7C638719780009279853%7CUnknown%7CTWFpbGZsb3d8eyJFbXB0eU1hcGkiOnRydWUsIlYiOiIwLjAuMDAwMCIsIlAiOiJXaW4zMiIsIkFOIjoiTWFpbCIsIldUIjoyfQ%3D%3D%7C0%7C%7C%7C&sdata=gSv4PhXCmD%2BptCudEIpvCoLSonjqqA0Anp%2BtagBBkkk%3D&reserved=0).

Reviewer #1: No

Reviewer #2: No

**Response: Thank you for your review.**
